# Influence of Nutrient Medium Composition on the Redistribution of Valuable Metabolites in the Freshwater Green Alga *Tetradesmus obliquus* (Chlorophyta) Under Photoautotrophic Growth Conditions

**DOI:** 10.3390/biotech14030060

**Published:** 2025-08-11

**Authors:** Elvira E. Ziganshina, Ayrat M. Ziganshin

**Affiliations:** Department of Microbiology, Institute of Fundamental Medicine and Biology, Kazan (Volga Region) Federal University, 420008 Kazan, Republic of Tatarstan, Russia

**Keywords:** *Tetradesmus obliquus*, photobioreactor, nutrient optimization, protein, starch, pigments

## Abstract

The study of microalgae has led to significant progress in recent decades. The current microalgal biomass yield is unsatisfactory, except for certain species that are cultivated for the nutraceutical and pharmaceutical industries. In this study, the growth efficiency and biochemical composition of *Tetradesmus obliquus* at high levels of nutrients were characterized. Increasing the NH_4_^+^-N content in the medium to 164 mg L^−1^ allowed the algae to steadily accumulate biomass (6.14 ± 0.28 g L^−1^) with a moderate content of starch. Optimizing the levels of N, P, and S allowed the biomass productivity to increase from the average 0.45 to 0.88 g L^−1^ day^−1^. A further increase of NH_4_^+^-N to 410 mg L^−1^ and other nutrients’ concentration allowed the algae to accumulate biomass (7.50 ± 0.28 g L^−1^), enriched with protein and pigments. The algae cultivated with the high load of nutrients reached 100%, 84%, and 96% removal of N, P, and S, respectively. Adding the NaHCO_3_ to the photobioreactor for pH adjustment (instead of NaOH) did not significantly improve the growth parameters or affect the composition of the algal cells. In general, our study will improve the comprehensive understanding of culture-based approaches to study the perspective use of the alga *T. obliquus*.

## 1. Introduction

Today, as for many millions of years, representatives of prokaryotic (Cyanobacteriota phylum, cyanobacteria) and eukaryotic microalgae (Bacillariophyta and Chlorophyta phyla, diatoms and green algae, respectively) form the basis of the aquatic and terrestrial ecosystems of the Earth. Microalgae have been widely studied as biotechnological agents due to their ability to produce a wide range of compounds of commercial interest [1,2]. Worldwide, many photosynthetic microorganisms are used as a source of valuable biomass (e.g., animal feed, fertilizers, plant stimulators) [2,3,4]; as biopharmaceuticals [5,6]; as agents for the biomonitoring, restoration, and conservation of ecosystems [7,8,9]; and as part of the carbon sequestration process [10]. However, despite long-term research programs aimed at developing microalgae-based biotechnologies and products, their cultivation faces limitations in industrial implementation [11,12].

Increased attention is being paid to individual metabolic products that are of greatest practical interest for agriculture, energy, nutraceutical, pharmaceutical, and cosmetic industries. Among the valuable algal compounds, lipids (polyunsaturated fatty acids), polysaccharides, proteins, essential amino acids, carotenoids, and vitamins should be noted [2,13]. Microalgae are being actively investigated to expand the list of antioxidants, anti-inflammatory, anticancer, antimicrobial, antiviral agents [5,6], and drug delivery systems [14]. Moreover, issues of including various microalgae in microbial consortia for the bioremediation of environmental objects are being actively addressed [15,16]. Despite considerable research in this area, only a few microalgal species are currently used to produce food, high-value chemicals, and to treat wastewater [11,12]. For example, members of the genus *Chlorella* are used to produce nutraceuticals [17,18], and the species *Haematococcus lacustris* (formerly called *Haematococcus pluvialis*) is used to produce the antioxidant astaxanthin [17,19].

The cultivation of microalgae is still at the conceptual stage due to several bottlenecks related to (i) the need to develop a cultivation strategy to achieve the maximum growth rate and nutrient uptake and (ii) the need to develop a biomass valorization strategy [12,20,21]. Today, scientific groups are developing different cultivation systems to overcome biological and non-biological limitations, as well as developing technical standards to stimulate the microalgal biotechnology industry. In this regard, knowledge of the intricacies of cultivation and information on the biochemical composition of valuable microalgal species will allow us to generate new ideas and expand the potential for the application of these phytoplankton objects in various industries [12]. Considering that the commercial significance of algae depends on their taxonomic status and physiological responses to biotic and abiotic factors, it is important to test promising strains under various growth conditions to determine the spectrum of their biotechnologically significant compounds and strategies for their production [22,23,24].

Members of the family *Scenedesmaceae* are among the most common green algae (phylum Chlorophyta) in freshwater environments. Despite the large volume of research, interest in the cultivation of representatives of the genera *Tetradesmus*, *Desmodesmus*, and *Scenedesmus* is only growing, and today many works are aimed at the development of effective cultivation methods [8,23,25]. Many studies focus on the integration of *Tetradesmus* species into carbon capture and/or wastewater treatment technologies, biofuels production [26,27], and biomonitoring as critical biomarkers of environmental changes [25,28]. The availability and concentration of essential chemical elements, pH, along with light and temperature, are key operating parameters to optimize the growth of microalgae. Many studies have reported that C and N sources shape the metabolic landscape of biotechnologically promising strains of the genus *Tetradesmus* [26,29], but the role of other key chemical elements such as P and S is often overlooked in such studies. *Tetradesmus obliquus* is a fast-growing microorganism that is being tested in the agricultural industry as a feed [30,31], for biofuel production [25,26,30], and as a candidate for applications in wastewater treatment [25,32]. This species can accumulate essential biomolecules [33] and maintain photosynthetic efficiency under N starvation stress conditions [34,35]. Thus, de Lira and colleagues [30] found that biomass of *T. obliquus* grown in 12 m^3^ photobioreactors with synthetic medium and diluted pig waste is very valuable because it contains a lot of volatile matter, fixed carbon, and protein. Authors highlighted the potential of this alga as a sustainable source for the bioenergy sector (solid biofuel and biodiesel) and industries focused on protein-rich raw materials. In another study [26], a rapid and energy-efficient method was developed to dry *T. obliquus* biomass grown under N-starved conditions in 16 m^3^ raceway ponds to produce high-quality biodiesel. In the study performed by Silva et al. [31], *T. obliquus* was reported not only as an alga with high protein, fiber, phenolic compounds, carotenoids, and essential fatty acids content but also as a safe food product with antioxidant activity.

A key aspect of studies to assess the potential of microalgae-based biotechnology, including waste and wastewater treatment processes, is the possibility of using products—microalgal biomass and their metabolites. The aim of this work was to determine the influence of macro- and microelement levels on the growth parameters of the native alga *T. obliquus*. Both the growth patterns and the excess nutrients on algal productivity were investigated, with prospects in various industries. Considering that algae are increasingly being introduced into the production of valuable biomass and into technologies for the bioremediation of polluted waters, the behavior of *T. obliquus* cells under conditions of excess nutrients is of scientific and practical interest.

## 2. Materials and Methods

### 2.1. Strain and Its Maintainence 

*Tetradesmus obliquus* strain B11 (hereafter *T. obliquus*) is from the algae culture collection of our laboratory at Kazan (Volga Region) Federal University. The alga was isolated from a freshwater reservoir (Kazan, Republic of Tatarstan, Russia; 55°79′ N, 49°09′ E) and then maintained on standard Bold’s basal medium (BBM; solidified with agar) with sodium nitrate as the N source (NO_3_^−^-N, 41 mg L^−1^) [36] and two antibiotics (10 µg of ampicillin and 50 µg of kanamycin per 1 mL of medium). The composition of the unmodified growth medium is given in Table 1.

The chloroplast *rbcL* gene encoding the large unit of ribulose-1,5-bisphosphate carboxylase/oxygenase (RuBisCO) was used for the molecular identification of the tested alga as described by us previously [37]. Polymerase chain reaction was performed using the primer pair rbcL-M379 F/rbcLFP R obtained from a published study [38], and the amplified fragments were sequenced using an Applied Biosystems 3130xl genetic analyzer (Thermo Fisher Scientific, Wilmington, DE, USA). The *rbcL* gene sequence showed 99.7% blast identity to *Tetradesmus obliquus*.

Before experiments in a photobioreactor, individual colonies from the solid BBM were transferred under aseptic conditions to 250 mL glass Erlenmeyer flasks containing 50 mL of BBM. The inoculation culture was grown in an orbital shaker–incubator at 120 rpm, and the cultivation period was 5 days. The average photosynthetic photon flux density (PPFD) (measured on the surface of the flasks) was 250 μmol photons m^−2^ s^−1^, which was provided by the Gro-Lux tubes. The growth temperature was maintained at 28 °C. The algal biomass was then pelleted at 3000× *g* for 5 min, was washed twice with sterile Na_2_HPO_4_ + KH_2_PO_4_ buffer (16 mM; pH 7.0), and was transferred into a 3.6 L Labfors 5 Lux autoclavable photobioreactor with a working volume of 2.6 L (Infors HT, Bottmingen, Switzerland). All experiments in the photobioreactor were started with an initial OD_750nm_ of 0.05 (optical density measured at 750 nm). The volume of inoculum used for cultivation in the photobioreactor ranged from 0.06% to 0.07% of the total medium volume. All manipulations with *T. obliquus* were carried out under aseptic conditions.

### 2.2. Cultivation in the Photobioreactor

The culture medium for the strain B11 grown in the photobioreactor was modified BBM with various reagents’ concentration (Table 2). The photobioreactor was operated under batch conditions and was illuminated by up to 16 Gro-Lux fluorescent tubes with high blue and red radiation (8 W and 120 lumen each). The present work was carried out by using a previously selected optimal external parameters for the algal growth and productivity [39], namely 24:0 (light/dark) photoperiod and high instantaneous PPFD, 1400 µmol photons m^−2^ s^−1^. In all experiments, the pH of the culture medium was strictly maintained at 7.0 ± 0.02. The experimental conditions for the growth of *T. obliquus* in all treatments are demonstrated in Table 2.

In experiments on the selection of the optimal load of macronutrients, NH_4_^+^-N was chosen as the main form of N based on our previous experiments with different green algae species [16,40]. This form of N that, when participating in N metabolism, entails lower energy losses for cells, whereas nitrate and nitrite ions require reduction [41]. Three series of experiments were conducted in this study. In the first set of experiments, the effects of increasing concentrations of N (as NH_4_^+^), P (as PO_4_^3−^), and S (as SO_4_^2−^) as the main macronutrients in the culture medium were investigated (Table 2, denoted as TR_1 (control)–TR_6). NH_4_Cl, K_2_HPO_4_/KH_2_PO_4_, and H_2_SO_4_ (c) were used to increase concentrations of N, P, and S. It is also worth noting that increasing the amount of PO_4_^3−^ also led to an increase in K^+^ levels. Thus, photoautotrophic experiments were carried out with six different N concentrations (standard and concentrations increased by two, four, six, eight, and ten times), with two P concentrations (standard and doubled concentrations), and with two S concentrations (standard and tripled concentrations). The pH stability (7.0 ± 0.02) in this set of experiments was ensured by an Infors system automatically supplying 2M NaOH (TR_1–TR_6). pH was measured in real time using an EasyFerm Plus PHI K8 425 electrode (Hamilton, OH, USA). The pressure in the reactor was controlled using a pressure transmitter 35XHT (Keller, Switzerland). The culture received a mixture of 2% CO_2_ and 98% air through a 0.20 µm Midisart filter (Sartorius Stedim Biotech, Göttingen, Germany) by bubbling. In the second set of experiments, the effect of doubling concentration of BBM reagents was investigated at elevated concentrations of N (in the form of 10 × NH_4_Cl and 10 × NaNO_3_), P, and S (designated as TR_7, TR_8, and TR_9). The inorganic C source in TR_7 and TR_8 was 2% CO_2_. The pH stability (7.0 ± 0.02) in this set of experiments was ensured by the Infors system automatically supplying 2 M NaOH in TR_7, 1 M HCl in TR_8, or 1 M NaHCO_3_ in TR_9. NaOH was replaced by NaHCO_3_ in TR_9 to assess the effectiveness of bicarbonate supply on the algal growth. Therefore, both CO_2_ and NaHCO_3_ were used as C sources in TR_9. A third set of experiments then focused on the feasibility of using NaHCO_3_ under conditions of favorable levels of N, P, and S under different CO_2_ regimens (TR_3, TR_10 – 2% CO_2_; TR_11, and TR_12 – ≈ 0.04% CO_2_). Foam control was achieved automatically by an Infors system delivering a sterile 2% antifoam B solution (Sigma-Aldrich, St. Louis, MO, USA). All treatments were repeated twice.

### 2.3. Assessment of Biomass and Its Composition

The growth, pigment concentration, and N consumption were measured daily. OD_750nm_ (a wavelength of 750 nm minimizes absorption by pigments [42]) was measured every day during the experiments using a UV/VIS spectrophotometer Lambda 35 (Perkin Elmer, Singapore) as the indicator of the algal culture density. Cell suspensions were diluted prior to measurements to obtain a final OD_750_ of less than 0.5. Additionally, the number of algal cells was estimated by using a counting chamber. Although many studies calculate biomass values based on the correlation between optical density and dry weight (DW) [43,44], in the present study, DW was measured empirically at the end of cultivation. The final DW (g L^−1^) and the level of volatile solids (VS, g L^−1^) of the biomass were analyzed using a drying oven and a muffle oven, respectively. Briefly, for the final weight measurements, the whole biomass was collected by centrifugation at 5000× *g* for 5 min. The final biomass was washed twice with distilled water. One part of the biomass was dried in a thermostat at +60 °C for 24 h and then used for protein and starch measurements. The other part was placed in crucibles and dried at +105 °C in a dry oven for 16 h (to determine the total solids or final dry matter). To estimate the VS in the algal biomass, crucibles were further placed in a muffle furnace at +550 °C for 2 h.

The specific growth rate (SGR, day^−1^), doubling time (DT, day), and biomass productivity (BP, g L^−1^ day^−1^) were calculated using the equations presented by Nayak et al. [44]. SGR was calculated during the exponential phase of growth (based on the OD_750nm_ values). BP was calculated by dividing the final DW by the total number of days within the experiment required to reach the stationary phase.

The chlorophylls *a*, *b*, and total carotenoids concentration was determined using the dimethyl sulfoxide extraction method as was described previously [45]. Briefly, 1 mL of the culture medium was centrifuged at 10,000× *g* for 5 min. The pellet was then treated with 1 mL of preheated solvent, resuspended, incubated for 5 min in a TS-100C thermo-shaker (BioSan, Riga, Latvia) at +60 °C, and then centrifuged at 10,000× *g* for 5 min. The absorbance of supernatant was measured at 480, 649, and 665 nm with the Lambda 35 spectrophotometer. The pigment contents were calculated using the equations given by Wellburn [46].

To measure protein and starch concentrations, algal samples were pretreated. For protein assessment, algal biomass (20 mg) dried at +60 °C and 1 mL of 0.5 M NaOH were added to a 2 mL metal lysing tube (MP Biomedicals, Illkrich, France) with two types of beads (0.6 g of zirconium/silicate beads of 0.1 mm in size and 0.4 g of glass beads of 1.0 mm in size). Homogenization was performed at a speed of 6.0 m s^−1^ for 30 s pulses twice with a cooling period of 5 min. Then, sample was incubated at 500 rpm in the TS-100C thermo-shaker at +80 °C. The mixture was then transferred into a clean tube and centrifuged at 2000× *g* for 5 min. The supernatant was used to measure protein content using a Bio-Rad protein assay kit (Munich, Germany) according to protocol. For starch assessment, the same algal biomass (50 mg), 0.2 mL of 80% (*v*/*v*) aqueous ethanol, and 0.8 mL of 1.7 M NaOH were added to the 2 mL metal lysing tube with the same two types of beads. Homogenization was performed at a speed of 6.0 m s^−1^ for 30 s pulses twice with a cooling period of 5 min. The mixture was then transferred to a clean glass test tube, and a starch assay kit K-TSTA (Megazyme, Germany) was used to quantify the total starch according to assay protocol.

### 2.4. Analytical Methods

Total ammonia nitrogen levels were measured using Nessler’s reagent (Sigma-Aldrich, Darmstadt, Germany), and the absorbance was measured at 425 nm using the Lambda 35 spectrophotometer. Nitrate, phosphate, and sulfate concentrations were measured using a Dionex ICS-900 ion chromatography system (Thermo Fisher Scientific, Wilmington, DE, USA) equipped with an IonPac AG22 (4 × 50 mm) guard column and an IonPac AS22 (4 × 250 mm) analytical column with the protocol described previously [47].

### 2.5. Statistical Analysis

Data for normal distribution were tested using the Kolmogorov–Smirnov test. Data were then statistically compared using the Tukey method and 95% confidence (Minitab software version 20.2.0, State College, PA, USA). All analyses were measured in triplicate, and the mean values are shown together with the standard deviations (error bars).

## 3. Results

### 3.1. Growth, Productivity, and Nutrient Uptake

*T. obliquus* was grown in the photobioreactor under controlled key process parameters (temperature, stirring, pH, aeration, CO_2_ supply, pressure in the culture vessel, and bright light conditions). The algae were grown in the modified BBM, the composition of which was changed to test the adaptation of the algae to a high nutrient level and to analyze the potential of the final biomass. Therefore, OD_750nm_, cell number, and DW values were used to characterize the growth of *T. obliquus*, while the levels of protein, starch, and pigments accumulated in the cells under different culture conditions were estimated to assess the commercial value of the tested microalgae. At the same time, monitoring the accumulation of pigments during algae cultivation was selected as an important indicator of the microalgal culture quality [11].

**Figure 1:**
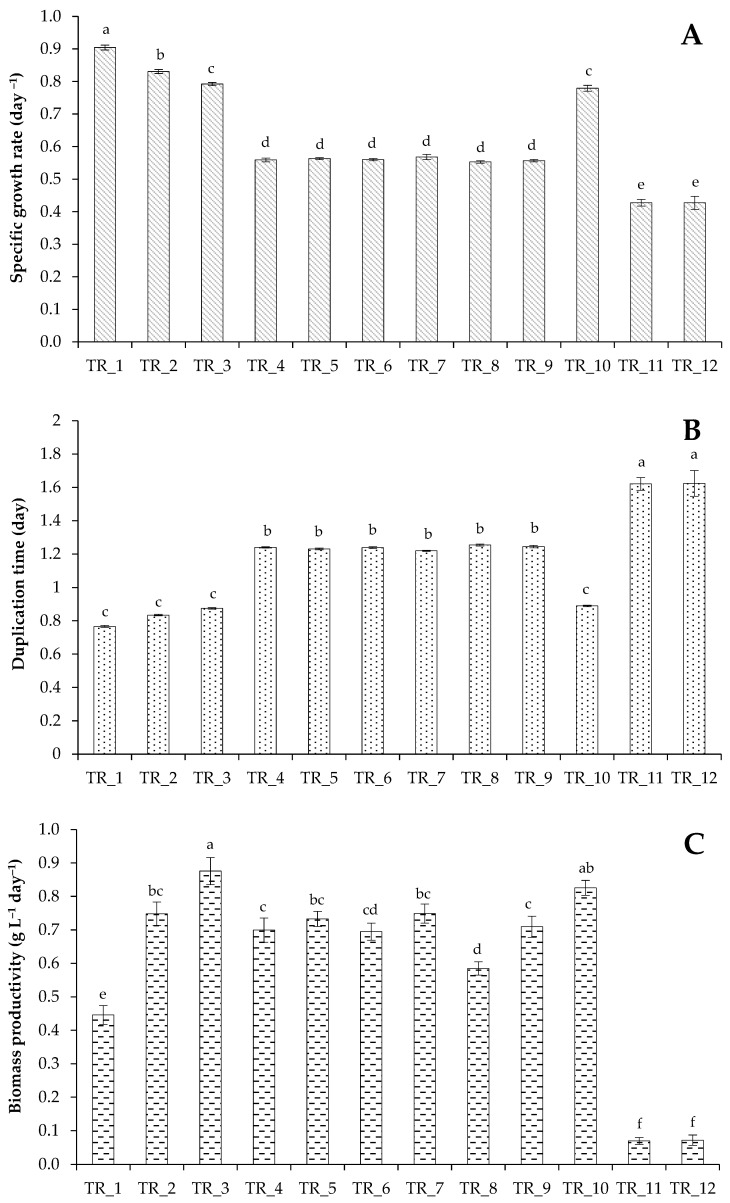
Specific growth rate (**A**), doubling time (**B**), and biomass productivity (**C**) of a culture of *T. obliquus* grown under strategies outlined in Table 2. Means that do not share a letter are significantly different (Tukey method and 95% confidence).

Culture characteristics, such as OD_750nm_, DW, VS, and final pigments, are shown in Table 3, whereas SGR, DT, and BP are illustrated in Figure 1. Multiple comparison analysis demonstrated significant differences in SGR values obtained in the control treatment (TR_1, with 41 mg L^−1^ of NH_4_^+^-N) compared to all other treatments with the modified BBM (Figure 1A). Increasing the concentration of ammonium N by two and four times in the presence of elevated levels of phosphate and sulfate ions in the medium (TR_2 and TR_3) allowed the microalgae to maintain relatively high average SGR values (0.79–0.83 day^−1^), but these values were lower than the values of the control treatment (0.90 ± 0.01 day^−1^; Figure 1A). Despite a slight decrease in SGR due to adaptation of algae to increased macronutrient content in the culture medium, the availability of additional macronutrients led to a statistically significant increase in DW (4.50 ± 0.21 g L^−1^ and 6.14 ± 0.28 g L^−1^ in TR_2 and TR_3, respectively). The average DW in the control experiments was at the level of 2.24 ± 0.14 g L^−1^ (Table 3).

A further increase in NH_4_^+^-N concentration (from 246 to 410 mg L^−1^) at sufficient concentrations of PO_4_^3−^ and SO_4_^2−^ (TR_4–TR_6) demonstrated a decrease in average SGR values up to 0.56 day^−1^ (Figure 1A). An increase in the N load in the nutrient medium (in the form of NH_4_Cl) extended the log phase that was reflected in the exit to the stationary growth phase. Although the modified nutritional conditions allowed for obtaining more biomass, this required a longer cultivation time (Table 3), which must be considered depending on the purpose of cultivation. Thus, if, with a four-fold increase in N concentration, the OD_750nm_ reaches maximum value after 7 days of cultivation, then to achieve the maximum OD_750nm_ with a further increase in the N level, the alga requires more than 10 days of cultivation.

The growth curves reflected the temporary adaptation of the alga *T. obliquus* to the increased content of inorganic forms of N, P, and S in the culture medium, expressed in similar or slightly reduced growth values during the first hours of cultivation. However, the final values of cell density were significantly higher than those obtained under growth conditions in a non-optimized growth medium. The increased N, P, and S concentrations had a positive effect on the BP values (Figure 1C), final biomass weight, and final pigment content (Table 3).

The obtained results demonstrated that increasing the concentration of N (as NH_4_^+^), P (as PO_4_^3−^), and S (as SO_4_^2−^) in the medium allowed us to obtain high biomass values (7.34 ± 0.23 g of dry biomass per liter and 6.77 ± 0.21 g of volatile solids per liter in TR_5). These chemical elements in combination with C are important macronutrients for cellular metabolism. Given that inorganic C concentration is a key factor in the cellular metabolism, we used CO_2_ as the sole C source and kept its input level (2%) constant in TR_1–TR_8.

Considering that elevated N, P, and S concentrations may cause the deficiency of other chemical elements essential for growth [48], the concentration of the reagents 2–15 of unmodified BBM (Table 1) was doubled in the TR_7 and TR_8 experiments. Two main forms of N, namely NH_4_^+^-N and NO_3_^−^-N, were tested in these treatments. Despite TR_7 having the highest OD_750nm_ value (14.7 ± 0.24) and final biomass yield (7.50 ± 0.28 g L^−1^), the SGR in this treatment did not exceed an average of 0.57 day^−1^, which can be explained by the acclimatization of the algae to the elevated values of macro- and microelement levels (Table 3; Figure 1). The absence of micronutrient deficiency in the culture medium with NH_4_^+^-N allowed the culture to slightly increase BP (0.75 ± 0.03 g L^−1^ day^−1^). Finally, more biomass was obtained in TR_7 over the same time period compared to TR_6. At the same time, cultivation in the presence of elevated level of NO_3_^−^-N (TR_8) resulted in a lower biomass yield (5.86 ± 0.20 g L^−1^). Although TR_4–TR_7 yielded high biomass levels, these experiments require longer cultivation times (Table 3), which is important to consider when analyzing the efficiency of commercial cultivation.

**Figure 2:**
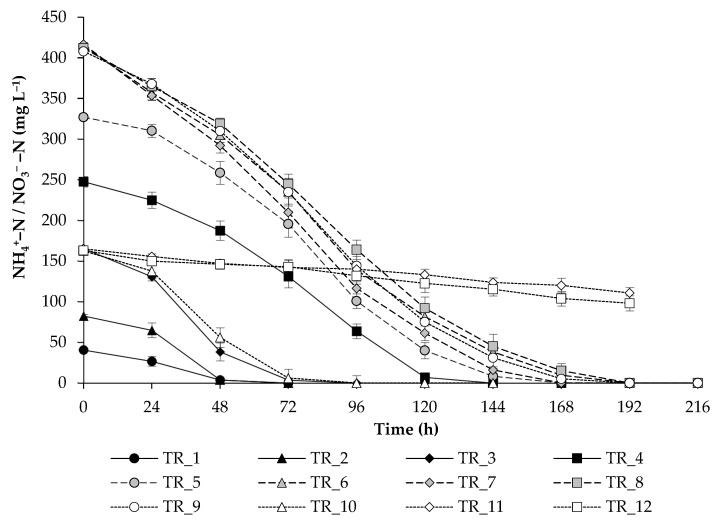
NH_4_^+^-N or NO_3_^−^-N utilization by a culture of *T. obliquus* grown under strategies outlined in Table 2.

**Figure 3:**
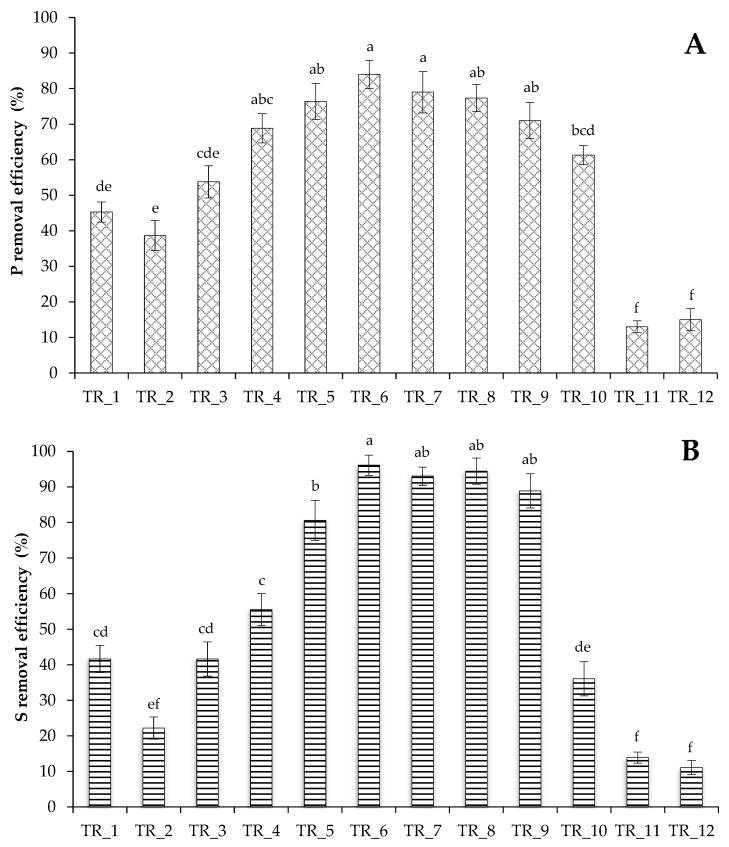
P (**A**) and S (**B**) removal efficiency by a culture of *T. obliquus* grown under strategies outlined in Table 2. Means that do not share a letter are significantly different (Tukey method and 95% confidence).

N was completely utilized in TR_1–TR_10 experiments (Figure 2). The algae resistant to high levels of N can be used to treat municipal and agricultural waste with high N loads. Phosphates were not completely utilized by the strain from the medium, which may indicate their sufficient level in the growth medium (Figure 3A). Maximum utilization was noted in TR_6 at high ammonium N load (84 ± 4% removal). The trends of sulfate utilization (Figure 3B) were similar to that of phosphate. Finally, the selected concentrations of PO_4_^3−^-P (106 mg L^−1^) and SO_4_^2−^-S (36 mg L^−1^) were sufficient for the alga.

The acidification of the culture medium in TR_1–TR_7 (H^+^ release during ammonium uptake by the alga) was prevented by a controlled addition of 2 M NaOH. NaOH was replaced by NaHCO_3_ in TR_9 to control pH level and to assess the effectiveness of bicarbonate supply on the algal growth. SGR, BP, DW, and nutrient removal in TR_9 were comparable to those observed in TR_7, indicating that the additional C source did not have a significant stimulatory effect on the algal growth.

The effects of several methods of inorganic C supply on the microalgal culture was tested in TR_10, TR_11, and TR_12 with the similar N load as in TR_3 (Table 2). The results showed that inorganic C form and concentration affected the growth of the culture. *T. obliquus* could effectively uptake inorganic C added as CO_2_ and efficiently utilize it for biomass production (TR_3). However, the addition of NaHCO_3_ (TR_10) did not allow the culture to show better growth kinetics (Figure 1). It should be noted that 16–17 mL of 1 M NaHCO_3_ per 1 L of a culture medium was added to the reactor to control pH level in TR_10 during active ammonium consumption. Finally, the algae grown in the modified medium supplemented with 2% CO_2_ (TR_3) had a higher SGR of 0.79 ± 0.01 day^−1^ compared to TR_10, TR_11, and TR_12 under similar experimental conditions but with different C regimens (0.77 ± 0.01 day^−1^, 0.43 ± 0.02 day^−1^, and 0.42 ± 0.03 day^−1^, respectively; Figure 1). The benefits of continuous CO_2_ supply were further confirmed by final DW and VS values (Table 3).

Thus, the increase in N concentration contributed to the increase in BP, which allowed the alga to utilize phosphate and sulfate ions more efficiently. The addition of nutrients, a continuous supply of 2% CO_2_, and the addition of NaOH to control the pH level of the synthetic nutrient medium (at 7.0 ± 0.02) allowed the alga to actively convert the provided inorganic compounds into organic matter.

### 3.2. Effect of Nutrient Loading on the Biochemical Composition of T. obliquus Cells

**Figure 4:**
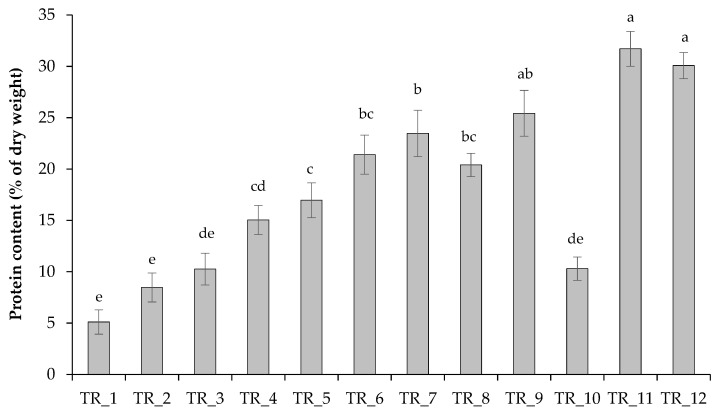
Protein content of a culture of *T. obliquus* grown under strategies outlined in Table 2. Means that do not share a letter are significantly different (Tukey method and 95% confidence).

**Figure 5:**
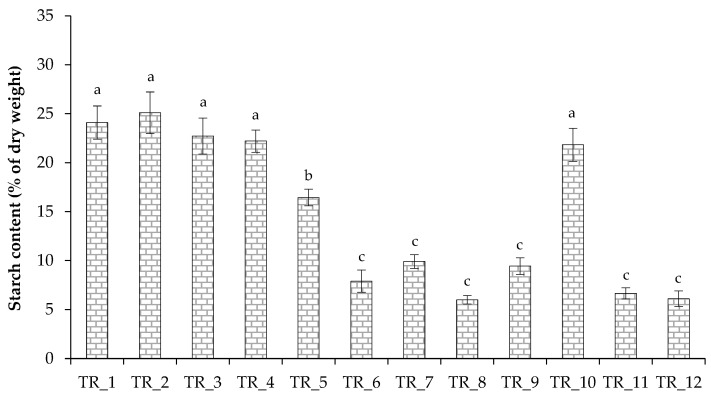
Starch content of a culture of *T. obliquus* grown under strategies outlined in Table 2. Means that do not share a letter are significantly different (Tukey method and 95% confidence).

**Figure 6:**
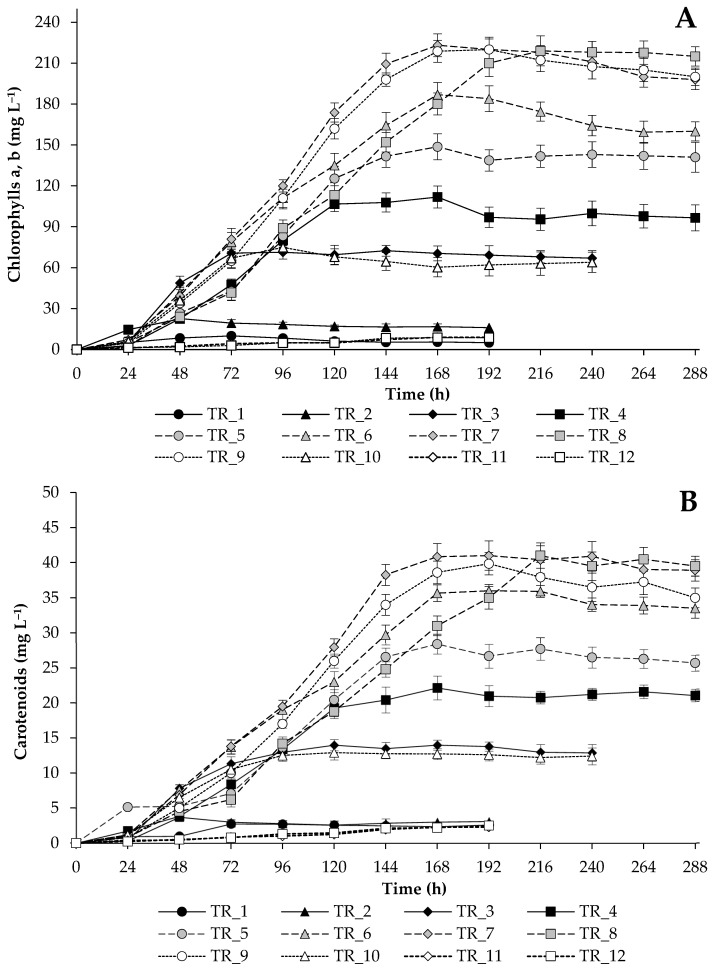
Total chlorophylls (**A**) and total carotenoids (**B**) concentrations obtained during the growth of *T. obliquus* under strategies outlined in Table 2.

To assess the effect of nutrient loading on the metabolism of *T. obliquus*, the content of protein, starch, and pigments in cells was compared. Under sufficient nutrient conditions, carbon fixed during photosynthesis was actively used by the algae for cellular proliferation and protein synthesis while not being actively spent on carbohydrate synthesis (Figure 4, Figure 5 and Figure 6). Nitrogen accounts for 1 to 14% of the DW of microalgae [41], and its external supply can stimulate the synthesis of proteins, pigments, and nucleic acids. In our case, the theoretical N content in experiments TR_1–TR_10 ranged from 1.82 to 6.99% of dry weight, and increasing the N level in the growth medium resulted in higher N contents in the cells.

Regarding the protein level, as expected, the increase in N concentration in the medium promoted the algal cells to accumulate protein (Figure 4). The protein content under the tested conditions ranged from 5.1% to 31.7% of DW. The level of protein in algal cells during growth in the modified medium increased and amounted to 21.4 ± 1.9% of DW with a ten-fold increase in N-concentration in the culture medium (TR_6), while an additional supply of micronutrients (TR_7 and TR_8) did not significantly affect the protein pool in the cells. Moreover, replacing NaOH with a controlled supply of NaHCO_3_ for pH control under similar nutrient loading conditions (TR_10 versus TR_3 and TR_9 versus TR_7) did not lead to a significant increase in soluble protein levels in the biomass. In experiments with air injection alone (≈400 ppmv CO_2_; TR_11) and in experiments with air injection and controlled NaHCO_3_ supply (TR_12), protein levels were also comparable (Figure 4).

Microalgae exposed to different stress factors, including nutritional ones, increase the intracellular content of reserve lipids, carbohydrates, and antioxidant enzymes [11]. Starch is the main form of energy and carbon storage in microalgal cells forming during photosynthetic CO_2_ fixation and provides energy for vital processes such as cell division [49]. The analysis of biochemical composition of algal cells under the tested conditions confirms that the tested *T. obliquus* accumulated protein and pigments in response to abundant N supply, without actively storing carbohydrates. Conversely, low N content in the growth medium led to a relatively high starch content in the algal cells and a decrease in N-containing metabolites. Thus, the algal cells with the high VS values (TR_7) were characterized by protein and starch contents of 23.5 ± 2.3% and 9.9 ± 0.7% of dry weight, respectively (Figure 4 and Figure 5).

The analysis of chlorophylls’ accumulation (Figure 6A) showed that the cells were provided with valuable N. It can be indirectly noted that the algal cells were not stressed, since they did not use the N of the pigments to synthesize enzymes necessary for cell viability under possible stress conditions. The total content of chlorophylls and carotenoids increased in cultures with increasing macronutrient loading, and high values were also achieved with additional micronutrient supplements (Figure 6, Table 3). Notably, when ammonium was replaced by nitrate ion, the rate of pigment accumulation was delayed, but relatively high values were obtained at the end of the experiment (4.37 ± 0.15% of DW). The replacement of NaOH with a controlled supply of NaHCO_3_ did not significantly affect the rate of accumulation and the level of pigments.

Finally, the abundance of inorganic carbon, nitrogen, phosphorus, and sulfur in combination with other chemical elements affected the growth and metabolism of *T. obliquus* culture. Therefore, to provide factors that ensure high biomass yield or to modulate the biochemical composition of microalgae according to its intended application, it is important to consider the contribution of both macronutrients and micronutrients and their combined effects.

## 4. Discussion

Microalgae are often cultured using culture media with fixed recipes, but there is growing evidence that culture media need to be adapted to the metabolic needs of the microalgae or to the cultivation goals. Therefore, research groups are increasingly focusing on a detailed understanding of the abiotic factors and nutrient supply responsible for the growth of commercially promising microalgae [48,50]. New data allow for expanded recommendations for the industrial production of algae-based value-added compounds, CO_2_ sequestration, and the development of biotechnologies using sustainable and competitive microalgae, for example for bioremediation applications [51,52]. This requires the research and development of innovative strategies for the commercialization of algae-based biotechnologies using sustainable and controlled cultivation, which is not possible under flask culture conditions.

In this study, the effects of different concentrations of reagents of the widely used standard synthetic medium on the growth of green algae were investigated in detail, and pH control strategies were compared in photoautotrophic controlled cultivation. Since during growth on ammonium the pH of the medium decreases, and during growth on nitrate the pH of the medium increases, in all experiments pH of the culture medium was strictly maintained at neutral conditions. Additionally, it should be noted that the photosynthetic activity of the algae (photoautotrophic mode) produces OH^−^, which gradually causes an increase in the pH of the culture medium. Changes in the pH, including changes through the consumption of other nutrients and the degradation of metabolites, can negatively affect the growth of microalgae, so pH control has been chosen as an important strategy to maintain efficient growth of microalgae. Based on the growth kinetics and productivity values of *T. obliquus* cells, it can be concluded that an increase in N (as NH_4_^+^) of up to four times with sufficient concentrations of P (as PO_4_^3−^) and S (as SO_4_^2−^) in the culture medium allowed the algae to stably accumulate biomass without significant influence to the growth rate, but cultivation in the presence of high ammonium concentration may affect some economic indicators due to the duration of cultivation. On the other hand, a ten-fold increase in N concentration in the culture medium did not have a toxic effect on the tested algae and was noted as non-inhibitory [53], which is attractive for bioremediation tasks when remediation agents resistant to environmental stress are needed.

Increasing the concentration of N, P, and S in the synthetic culture medium allowed us to obtain high algal biomass values (7.34 ± 0.23 g L^−1^ DW and 6.77 ± 0.21 g L^−1^ VS in TR_5), since these chemical elements are important macronutrients for the cellular metabolism. The modification of the culture medium resulted in biomass production by *T. obliquus* B11 that was significantly higher than that obtained in other works conducted with this species. For example, in a study investigated the growth and lutein production by five *T. obliquus* strains cultured in a 1 L reactor in Detmer’s medium, the biomass yield ranged from 2.23 to 3.38 g L^−1^, and BP of the strains did not exceed 0.5 g L^−1^ day^−1^ [54]. In another study investigated the effects of elevated N supplementation on the growth and metabolite profiles of *T. obliquus*, the author found that changes in N concentration in BG-11 medium did not result in biomass production greater than 1.32 g L^−1^ [55]. Another species of this genus, *Tetradesmus wisconsinensis*, cultured in a 1 L Erlenmeyer flask with 600 mL BBM under different inorganic C contents, had a maximum growth rate and final cell DW of 0.33 day^−1^ and 0.67 g L^−1^, respectively [56]. N is an essential component for photosynthesis, respiration, proteins, and enzymes, and microalgae can consume increased concentrations of N as well as P to increase the synthesis of proteins and nucleic acids [13,48,53]. However, in many studies, factors such as light, temperature, and pH remain underestimated. It is also worth noting several works aimed at studying the effects of different N sources and their concentrations to determine the optimal level for accumulation of energy storage compounds, such as lipids, in response to the increasing levels of cellular oxidative stress in microalgal cells [52,57,58]. In these cases, the researchers mainly studied the productivity characteristics of algal cultures in N-deficient media, whereas in the present work the culture was provided with a high concentration of N.

Providing cells with a reduced form of N, ammonium, allows energy to be distributed to such important cellular processes as photosynthesis, synthesis, and accumulation of nitrogenous compounds, mainly, proteins, and chlorophylls [59]. Thus, the addition of NH_4_^+^-N allowed us to obtain more attractive values for both growth kinetics and the biochemical composition of *T. obliquus* cells compared to the growth on NO_3_^−^-N. Microalgal strain *T. obliquus* B11 cultured in the photobioreactor under photoautotrophic conditions with the highest load of nutrients (NH_4_^+^-N, PO_4_^3−^-P, SO_4_^2−^-S: 410, 106, and 36 mg L^−1^, respectively) and with doubled levels of other components of BBM resulted in attractive values of total biomass compared to the values when grown on nitrates (TR_7 versus TR_8). These results are in line with previous studies that found slower growth rates when microalgae were grown on nitrate-containing culture media compared to ammonium-containing media. This is due to the fact that microalgae use energy to reduce NO_3_^−^ and produce nitrate and nitrite reductases [41].

P also plays an important role in the energy balance and is important for the structure of membranes and nucleic acids; its limitation reduces the rate of photosynthesis and growth of cells. Its content is within 1% of the total biomass of algae, and algae can assimilate P (in the form of orthophosphate and polyphosphate) at higher concentrations than required for the growth and use it to convert into ATP under unfavorable nutritional conditions [13]. P and S removal efficiency indicate that the increase in their concentrations (up to 106 and 36 mg L^−1^, respectively) was sufficient for the algal growth. The present study shows that the increase in phosphate and sulfate in the culture medium at elevated N content was sufficient for biomass accumulation, and the ability of algae to actively utilize the provided macronutrients is attractive for its application in the bioremediation industry. Some studies have shown that high levels of macronutrients can cause toxic effects and reduce the growth performance of microalgae. Thus, in a recent study devoted to the cultivation of microalgae for the development of space missions, growth-limiting levels of C, N, and P were determined for *Chlorella vulgaris* [60].

Based on obtained data, NaHCO_3_, which was served as the additional C source and tested instead of NaOH to control pH level, had no sufficient stimulatory effect on the alga. These results are not consistent with studies in which the authors suggested bicarbonate as a promising C nutrient, but it is important to note that pH was not controlled in these experiments and nitrate served as the main source of N [56,61]. pH significantly influences the content of carbon forms in the growth medium. As pH increases, CO_3_^2−^ concentrations increase and HCO_3_^−^ and aqueous CO_2_ concentrations decrease, and vice versa. In the present study, HCO_3_^−^ was the predominant form of dissolved inorganic C at a controlled neutral pH. The obtained results can also be explained by the fact that the addition of NaOH to the culture medium to maintain pH enhances the retention of CO_2_ in the culture medium [62,63], and C was successfully utilized by microalgae. It is also worth considering that the active absorption of CO_2_ is energetically more favorable than HCO_3_^−^ [64]. The bicarbonate-based cultivation of microalgae is considered more practical [65], but several studies on the use of NaHCO_3_ as inorganic C source note that NaHCO_3_ can delay growth, including due to the increased concentration of sodium ions in the growth culture [66,67]. Therefore, it is necessary to determine the optimal cultivation conditions with bicarbonate for each promising candidate. At the same time, the cultivation of algae with CO_2_ addition can solve ecological issues, what emphasizes the increase in studies on the use of different industrial gases in various industries [68,69].

The biochemical composition of microalgae is species-dependent and varies according to the operating parameters of the bioreactors and composition of the nutrient medium. Microalgae can store excess nutrients, particularly in the form of sugars, proteins, lipids, and pigments [11]. A relatively large number of works are devoted to the cultivation of microalgae, including species of *Tetradesmus* genus, under conditions of N starvation. The authors note the growth inhibition, but at the same time an increase in the levels of carbohydrates and fatty acids (which are stored in the cells of microalgae as sources of C and energy) [13,56,70]. A recent study examined the effects of inorganic C sources on biomass and lipid production by *T. wisconsinensis*, a candidate for biodiesel production [56]. In this work, the biochemical differences between biomass of *T. obliquus* grown in standard and modified BBM were described. *T. obliquus* efficiently accumulated pigments and protein, while the proportion of starch decreased in response to the optimization of the growth medium. High pigment levels (up to 4.37% of dry mass) and high protein content (up to 32% of dry mass) in *T. obliquus* cells open prospects for its industrial use. *T. obliquus* was assessed in the work by Cabrita et al. [71] as a product with a rich amino acid profile that can compensate for the deficiency of amino acids in cereal-based diets. This allowed the authors to classify this microorganism as a product with health benefits for animals. Another study evaluated the introduction of high doses of the microalga *T. obliquus* to the diet of Wistar rats. The diet containing microalgae was noted to have positive effects on animal growth and health [31].

The selected optimal range of nutrient media composition will allow it to move in the future from laboratory-scale experiments to industrial production, and information on the distribution of metabolites in cells and the maximum level of their accumulation will allow the further testing of extraction methods. It is worth noting that the main emphasis is currently placed on methods that allow achieving not only the high purity of the product but also methods with a low impact on the environment. Subcritical water extraction and supercritical fluid extraction are promising extraction methods that have demonstrated their efficiency in recovering high-value compounds from microalgae. These methods have high selectivity and a short extraction time and do not require the use of toxic organic solvents, while green solvents, i.e., biodegradable, non-toxic solvents obtained from natural sources (for example, terpenes from citrus fruits), have also been extensively studied [72].

In the present study, the starch level in microalgal cells during photoautotrophic cultivation under various conditions was assessed. Since nutrient limitation, particularly N or P, usually results in an increase in the C content of biomass [48], a decrease in the proportion of starch is expected in cells grown in a culture medium with elevated nutrient level. The authors of a study aimed at assessing the morphology and physiology of *T. obliquus* BR003 of tropical origin grown under standard conditions noted that the strain’s chloroplasts had many starch granules [33]. In the present study, the increase in available N promoted the accumulation of protein in algal cells, and we propose that by increasing proteins and carotenoids as antioxidants [73] the alga actively adapted to a high nutritional regimen and was not forced to store starch as energy and C reserve. Finally, the controlled cultivation of *T. obliquus* revealed that relatively low levels of nutrient loading were suitable for starch accumulation in the cells, while higher nutrient concentrations improved biomass yield and affected the partitioning of the algal biochemical components towards pigments and proteins, which is useful for potential biotechnological applications. The obtained data are also useful for the ecology industry, since algae in their natural habitats as the first organisms in the food chain face many environmental stresses, including those mediated by the eutrophication of water bodies.

## 5. Conclusions

The physiological and biochemical responses of the promising alga *Tetradesmus obliquus* B11 were studied when it was given more macronutrients and micronutrients than generally accepted levels. Biomass indices, obtaining and maintaining a high level of chlorophylls, and the absence of their degradation throughout the experimental period made it possible to reveal the normal physiological state of the tested photosynthetic organism under high nutritional load conditions. The algae demonstrated high adaptability to increasing levels of essential nutrients in the culture medium and suitability for bioremediation purposes. The highest biomass concentration was achieved when the algae were axenically cultivated in the medium with 410 mg NH_4_^+^–N L^−1^, 106 mg PO_4_^3−^-P L^−1^, and 36 mg SO_4_^2−^-S L^−1^, whereas increasing N in the nutrient medium by up to four times allowed the algae to steadily accumulate biomass without significant damage to the growth rate. Selecting a pH regulation strategy demonstrated that the supply of NaHCO_3_ did not provide an increase in the main growth parameters of the culture and did not affect the distribution of the main metabolites. The obtained results will increase the awareness of microalgal cultivation under controlled conditions in photobioreactors, including commercial applications. The practical significance of the work lies in the selection of controlled cultivation conditions to increase the productivity and commercial attractiveness of biomass.

## Figures and Tables

**Table 1:** Composition of standard BBM.

#	Reagent	Final Concentration, mg L^−1^
1	NaNO_3_	250
2	MgSO_4_ 7H_2_O	75
3	NaCl	25
4	K_2_HPO_4_	75
5	KH_2_PO_4_	175
6	CaCl_2_ 2H_2_O	25
7	ZnSO_4_ 7H_2_O	8.82
8	MnCl_2_ 4H_2_O	1.44
9	Na_2_MoO_4_ 2H_2_O	1.19
10	CuSO_4_ 5H_2_O	1.57
11	Co(NO_3_)_2_ 6H_2_O	0.49
12	H_3_BO_3_	11.42
13	C_10_H_14_N_2_Na_2_O_8_ 2H_2_O	63.68
14	KOH	31
15	FeSO_4_ 7H_2_O	4.98

**Table 2:** Experimental conditions applied in the present manuscript.

Treatment	NH_4_^+^-N, mg L^−1^	NO_3_^−^-N, mg L^−1^	PO_4_^3−^-P, mg L^−1^	SO_4_^2−^-S, mg L^−1^	Inorganic Carbon Source	
					Continuous Supply of CO_2_, %	Controllable Supply of 1 M NaHCO_3_
TR_1	41	0.05	53	12	2.0	−
TR_2	82	0.05	106	36	2.0	−
TR_3	164	0.05	106	36	2.0	−
TR_4	246	0.05	106	36	2.0	−
TR_5	328	0.05	106	36	2.0	−
TR_6	410	0.05	106	36	2.0	−
TR_7 *	410	0.1	106	36	2.0	−
TR_8 *	0	410.1	106	36	2.0	−
TR_9 *	410	0.1	106	36	2.0	+
TR_10	164	0.05	106	36	2.0	+
TR_11	164	0.05	106	36	~0.04	−
TR_12	164	0.05	106	36	~0.04	+

Maintained parameters for all treatments: 24 h photoperiod; instantaneous PPFD (1400 µmol m^−2^ s^−1^); temperature (+28 °C); pH (7.0 ± 0.02); aeration (0.7 L min^−1^); stirring (120 rpm); pressure in the culture vessel (30 ± 2 mBar). * In the experiments TR_7, TR_8, and TR_9, the concentration of the components 2–15 of standard BBM (Table 1) was doubled.

**Table 3:** Characteristics of microalgae cultivated under different conditions.

Treatment	Final OD_750nm_	Final Dry Weight, g L^−1^	Volatile Solids, g L^−1^	Final Pigments, % of Dry Weight	Enter to the Stationary Phase, Day	Period of Cultivation, Day
TR_1	4.6 ± 0.17 ^f^	2.24 ± 0.14 ^e^	2.14 ± 0.14 ^e^	0.34 ± 0.02 ^f^	5	8
TR_2	7.3 ± 0.21 ^e^	4.50 ± 0.21 ^d^	4.34 ± 0.19 ^d^	0.42 ± 0.02 ^f^	6	8
TR_3	12.8 ± 0.20 ^c^	6.14 ± 0.28 ^bc^	5.92 ± 0.27 ^bc^	1.30 ± 0.06 ^e^	7	10
TR_4	13.4 ± 0.25 ^bc^	7.01 ± 0.36 ^ab^	6.75 ± 0.33 ^ab^	1.68 ± 0.09 ^de^	10	12
TR_5	14.0 ± 0.32 ^ab^	7.34 ± 0.23 ^a^	6.77 ± 0.21 ^ab^	2.27 ± 0.07 ^cd^	10	12
TR_6	13.5 ± 0.31 ^bc^	6.96 ± 0.25 ^ab^	6.40 ± 0.23 ^abc^	2.78 ± 0.10 ^bc^	10	12
TR_7	14.7 ± 0.24 ^a^	7.50 ± 0.28 ^a^	7.21 ± 0.24 ^a^	3.16 ± 0.12 ^b^	10	12
TR_8	12.6 ± 0.35 ^cd^	5.86 ± 0.20 ^c^	5.60 ± 0.19 ^c^	4.37 ± 0.15 ^a^	10	12
TR_9	13.1 ± 0.21 ^bc^	7.11 ± 0.32 ^a^	6.81 ± 0.30 ^ab^	3.31 ± 0.15 ^b^	10	12
TR_10	11.7 ± 0.30 ^d^	5.79 ± 0.16 ^c^	5.58 ± 0.15 ^c^	1.32 ± 0.04 ^e^	7	10
TR_11	1.0 ± 0.07 ^g^	0.50 ± 0.07 ^f^	0.47 ± 0.07 ^f^	2.26 ± 0.32 ^cd^	7	8
TR_12	1.0 ± 0.14 ^g^	0.52 ± 0.11 ^f^	0.49 ± 0.09 ^f^	2.22 ± 0.42 ^cd^	7	8

Means that do not share a letter are significantly different according to the Tukey method and 95% confidence.

## Data Availability

The original contributions presented in this study are included in the article. Further inquiries can be directed to the corresponding authors.
